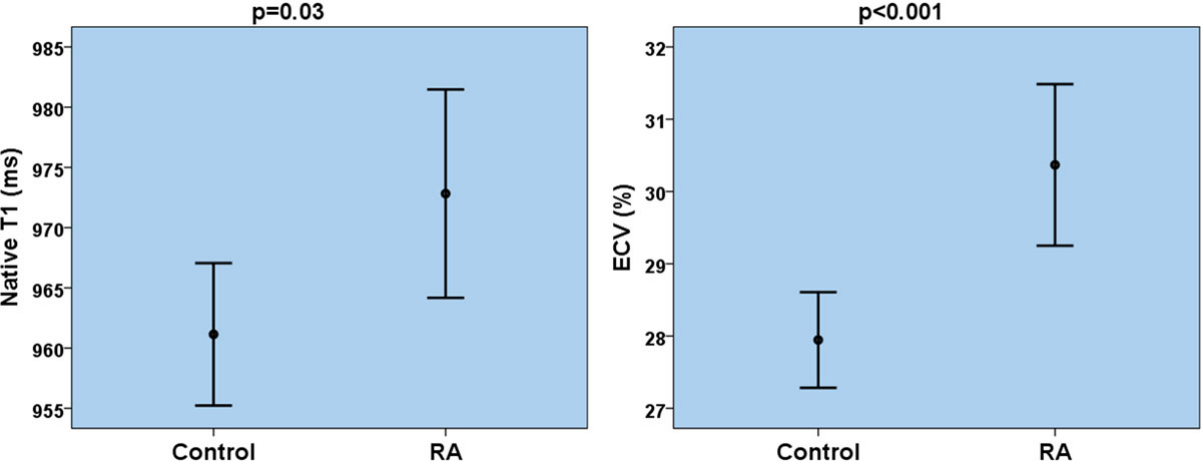# Diffuse myocardial fibrosis is associated with impaired myocardial strain and disease activity in rheumatoid arthritis: a cardiovascular magnetic resonance study

**DOI:** 10.1186/1532-429X-16-S1-P292

**Published:** 2014-01-16

**Authors:** Ntobeko A Ntusi, Stefan K Piechnik, Jane M Francis, Vanessa M Ferreira, Paul M Matthews, Matthew D Robson, Paul B Wordsworth, Stefan Neubauer, Theodoros D Karamitsos

**Affiliations:** 1 Division of Cardiovascular Medicine, Radcliffe Department of Medicine, University of Oxford & John Radcliffe Hospital, Oxford, UK; 2 GlaxoSmithKline Clinical Imaging Centre, GlaxoSmithKline, London, UK; 3 Division of Brain Sciences, Department of Medicine, Imperial College, London, UK; 4 NIHR Oxford Musculoskeletal Biomedical Research Unit & Nuffield Department of Orthopaedics, Rheumatology and Musculoskeletal Sciences, University of Oxford & Nuffield Orthopaedic Centre & John Radcliffe Hospital, Oxford, UK

## Background

Rheumatoid arthritis (RA) is a chronic autoimmune disease of the joints, with frequent extra-articular complications including cardiovascular disease and cardiac fibrosis from multiple causes. Diffuse myocardial fibrosis can be detected non-invasively by extracellular volume (ECV) mapping based on pre- and postcontrast T1 measurements using cardiovascular magnetic resonance (CMR). We therefore hypothesized that CMR T1 mapping can detect subclinical diffuse myocardial fibrosis in patients with RA.

## Methods

39 RA patients (28 female, mean age 50 ± 12 years) and 39 matched controls (28 female, mean age 49 ± 12 years) without previously known cardiovascular disease underwent CMR at 1.5T. CMR assessments included late gadolinium enhancement (LGE) [IV gadoterate meglumine at 0.15 mmol/kg], T1 mapping, cine, tagging, and T2-weighted imaging.

## Results

Focal fibrosis on LGE was found in 18 (46%) RA patients compared to none of controls. Two of these patients had evidence of subendocardial enhancement, in keeping with previously unknown myocardial infarction. Evidence of diffuse myocardial fibrosis in RA was supported by higher precontrast T1 values (973 ± 27 vs. 961 ± 18 ms, p = 0.03), lower postcontrast T1 values (450 ± 40 vs. 468 ± 32 ms, p = 0.04) and expansion of ECV (30.3 ± 3.4 vs. 27.9 ± 2.0 %, p < 0.001) - the 2 RA patients with MI were excluded from this analysis. Indices of diffuse myocardial fibrosis were significantly elevated in RA, regardless of the presence of LGE. There was no difference in left ventricular volumes, mass and ejection fraction between RA patients and controls. However, there were differences in regional function: peak systolic circumferential strain (-16.9 ± 1.3 vs. -18.7 ± 1.2, p < 0.001) and peak diastolic strain rate (83 ± 21 vs. 112 ± 20 s-1, p < 0.001) were impaired in RA patients. Indices of diffuse myocardial fibrosis correlated with impaired myocardial systolic strain, diastolic strain rate and RA disease activity. There was no evidence of myocardial edema in RA.

## Conclusions

Cardiac involvement is common in RA patients with no cardiovascular symptoms, and includes both focal and diffuse myocardial fibrosis, which is associated with impaired systolic and diastolic strain parameters, as well RA disease activity. CMR is a robust non-invasive tool for the assessment of diffuse myocardial fibrosis, and may be useful in the follow-up of patients. Alterations in diffuse myocardial fibrosis likely precede significant changes in cardiac structure and function.

## Funding

This study was funded by an investigator-led grant from GSK to Dr. Theo Karamitsos. The authors gratefully acknowledge support from the National Institute for Health Research Oxford Biomedical Research Centre Programme. Prof. Stefan Neubauer also acknowledges support from the Oxford British Heart Foundation Centre for Research Excellence.

**Table 1:** Baseline characteristics and CMR findings

	Controls N = 39	RA N = 39	P value
Female sex, n (%)	28 (72)	28 (72)	1.00

Age, years	49 ± 12	50 ± 12	0.65

Hypertension, n (%)	2 (5)	5 (13)	0.65

Diabetes, n (%)	0	1 (3)	-

Hyperlipidaemia, n (%)	5 (13)	6 (15)	0.75

BMI, kg/m2	24 ± 4	26 ± 5	0.13

DAS28-CRP (median, IQR)	N/A	3.3 ± 1.3	-

ESR, mm/hr (median, IQR)	N/A	15 (9-19)	-

CRP, mg/L (median, IQR)	1 (1-2)	9 (4-13)	< 0.001

Hemoglobin, g/L	13 ± 1	13 ± 1	0.47

Duration of RA, years (median, IRQ)	N/A	7 (4-11)	-

Duration of DMARDs, years (median, IQR)	N/A	4 (3-6)	-

LVEDV indexed to BSA, ml/m2	78 ± 15	80 ± 16	0.63

LVESV indexed to BSA, ml/m2	24 ± 16	23 ± 9	0.62

LVEF, %	73 ± 5	72 ± 7	0.25

LV Mass indexed to BSA, g/m2	52 ± 11	54 ± 12	0.34

LA size, mm	27 ± 5	32 ± 5	< 0.001

Mid SA circumferential strain	-19.0 ± 1.2	-17.1 ± 1.2	< 0.001

Peak diastolic circumferential strain rate (s-1)	117 ± 18	85 ± 18	< 0.001

Presence of LGE (%)	0	18 (46)	-

Volume fraction of LGE > 2SD (%)	0	3.7 ± 0.4	-

STIR T2 Ratio	1.5 ± 0.1	1.6 ± 0.2	0.07

Continuous data are mean ± SD unless otherwise indicated. BMI, body mass index; CRP, C-reactive protein; DMARD, disease modifying anti-rheumatic drug; ESR, erythrocyte sedimentation rate; IQR, interquartile range; LA, left atrium; LGE, late gadolinium enhancement; LV, left ventricle/ventricular; LVEDV, left ventricular end-diastolic volume; LVEF, left ventricular ejection fraction; LVESV, left ventricular end-systolic volume; RA, rheumatoid arthritis; SA, short axis; STIR, short Tau inversion recovery

**Figure 1:**